# Optical Method for Cardiovascular Risk Marker Uric Acid Removal Assessment during Dialysis

**DOI:** 10.1100/2012/506486

**Published:** 2012-05-22

**Authors:** Jana Holmar, Ivo Fridolin, Fredrik Uhlin, Kai Lauri, Merike Luman

**Affiliations:** ^1^Department of Biomedical Engineering, Technomedicum, Tallinn University of Technology, Ehitajate tee 5, EST-19086 Tallinn, Estonia; ^2^Department of Medicine and Health Sciences, Faculty of Health Sciences, Linköping University, SE 581 85 Linköping, Sweden; ^3^Department of Nephrology UHL, County Council of Östergötland, SE 581 85 Linköping, Sweden; ^4^Centre of Nephrology, North Estonian Medical Centre, Tallinn, Estonia

## Abstract

The aim of this study was to estimate the concentration of uric acid (UA) optically by using the original and processed ultraviolet (UV) absorbance spectra of spent dialysate. Also, the effect of using several wavelengths (multi-wavelength algorithms) for estimation was examined. This paper gives an overview of seven studies carried out in Linköping, Sweden, and Tallinn, Estonia. A total of 60 patients were monitored over their 188 dialysis treatment procedures. Dialysate samples were taken and analysed by means of UA concentration in a chemical laboratory and with a double-beam spectrophotometer. The measured UV absorbance spectra were processed. Three models for the original and three for the first derivate of UV absorbance were created; concentrations of UA from the different methods were finally compared in terms of mean values and SD. The mean concentration (micromol/L) of UA was 49.7 ± 23.0 measured in the chemical laboratory, and 48.9 ± 22.4 calculated with the best estimate among all models. The concentrations were not significantly different (*P* ≥ 0.17). It was found that using a multi-wavelength and processed signal approach leads to more accurate results, and therefore these approaches should be used in future.

## 1. Introduction

Uric acid (UA), a final product of the metabolism of purine, is a very important biological molecule present in body fluids. It is mostly excreted from the human body through the kidneys in the form of urine. The concentration of UA in blood increases when the source of UA increases or the kidneys malfunction. Hyperuricemia is a symptom when the UA concentration is above 7 mg/dL. UA is hard to dissolve in blood and will crystallise when supersaturated. The UA crystallites are deposited on the surface of the skin, in joints, and particularly in the toes, resulting in gout. Analysis of the UA concentration in blood helps to diagnose gout. In addition to gout, hyperuricemia is connected with lymph disorders, chronic haemolytic anaemia, an increase in nucleic acid metabolism, and kidney malfunction. Elevated serum UA contributes to endothelial dysfunction and increased oxidative stress within the glomerulus and tubulointerstitium, with associated increased remodelling fibrosis of the kidney [1]. A high level of serum UA, hyperuricemia, has been suggested as an independent risk factor for cardiovascular and renal diseases [2] especially in patients with heart failure, hypertension, and/or diabetes [3–5], and has been shown to cause renal disease in a rat model [6]. UA is mostly associated with gout, but studies have implied that UA affects biological systems [7] and could also influence the risk of higher mortality among dialysis patients [8], although the pathogenic role of hyperuricemia in dialysis patients has not been fully established [9]. High caloric foods and alcohol as well as disorders of the organs and tissues are the main causes of hyperuricaemia, obesity, kidney stone formation, and even gout [10]. It is likely that high UA levels in the blood are the reason for the emergence of renal microvascular disease, which may be a key mechanism in inducing salt-sensitive hypertension [11]. Harm can be prevented and reduced by early diagnosis and monitoring, especially by screening obese patients [12].

It would be advantageous to measure the concentration of UA during dialysis online. For creating this opportunity it is necessary to create accurate and reliable models. UA may be the novel marker molecule for estimating the quality of dialysis procedure, since the UA is uremic toxin itself, removal pattern and amount of this compound during the dialysis are informative for patients and medical personnel.

Ways of monitoring UA, dialysate, and other biological fluids with optical tools have been shown previously by our and other groups [13–15]. If you use a simple signal processing tool for smoothing and calculating the first derivate of UV absorbance and/or absorbance or processed absorbance values from several wavelengths, more reliable results are achieved [16–20]. An effective way of estimating UA concentrations using the UV technique has been shown in previous studies by our group. Current paper, involving larger amount of patients from different countries, presents more general and accurate models making it possible to apply the technique in the large patient community.

The aim of this study was to estimate the concentration of uric acid (UA) optically by using the original and processed ultraviolet (UV) absorbance spectra of spent dialysate. Data from different dialysis centres and over a long period was used to build models to increase general validity and reliability.

## 2. Materials and Methods

All of the studies were performed after approval of the protocol by the Regional Ethical Review Board, Linköping, Sweden, and by the Tallinn Medical Research Ethics Committee at the National Institute for Health Development, Estonia. Informed consent was obtained from all participating patients.

**Figure 1:**
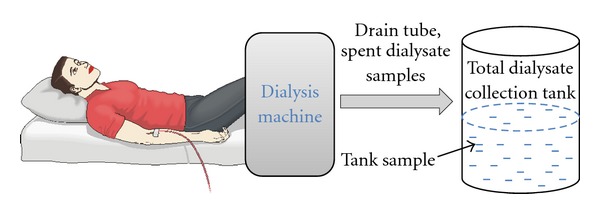
Schematic clinical setup of the experiments.

During the period 1999–2009 seven studies were carried out in the Department of Dialysis and Nephrology at the Linköping University Hospital in Sweden and at the North Estonian Medical Centre in Estonia. Clinical setup of the experiments is presented in Figure 1. A summary of the studies and information about the participating patients are presented in Table 1.

The dialysers used in the studies, the effective membrane areas of the dialysers, the number of sessions when the respective dialyser was used, the type of dialysis machine used, and blood flow for the studies are presented in Table 2.

For all of the studies, samples of spent dialysate were taken at discrete times for analysis (Table 3). The numbers under “sampling time” correspond to the number of minutes after the start of hemodialysis. The dialysate samples were taken at 255, 270, and 300 minutes when the duration of sessions was long enough. Also, the sample from the total dialysate collection tank was included in the analysis in most cases. Pure dialysate was collected before the start of a dialysis session and used as the reference solution when the dialysis machine was prepared and conductivity was stable.

The concentration of UA was determined in the Clinical Chemistry Laboratories at the North Estonian Medical Centre and at Linköping University Hospital using standardised methods. The accuracy of the methods for the determination of UA in dialysate was ±5%.

Double-beam spectrophotometers (UVIKON 943, Kontron, Italy, and JASCO V-570, UV/VIS/NIR spectrophotometer, Japan, in Linköping and SHIMADZU UV-2401 PC, Japan, in Tallinn) were used for the determination of UV absorbance. Spectrophotometric analysis over a wavelength range of 190–380 nm was performed by an optical cell with an optical path length of 1 cm. A lower UV absorbance value is obtained at all wavelengths versus time due to a decreased concentration of UV-absorbing compounds in the blood when transported through the dialyser into the dialysate and removed from the blood during the dialysis treatment. The treatments were also monitored with a single wavelength online, and thereby all interruptions, self-tests, alarms, and so forth could be identified directly on a screen. Some of the measured values (absorbance or concentration) were excluded from data before analysis. The exclusion criteria were incorrect or illogical values of measured concentration or absorption, for example, sampling coexisting with self-tests of the dialysis machine.

**Figure 2:**
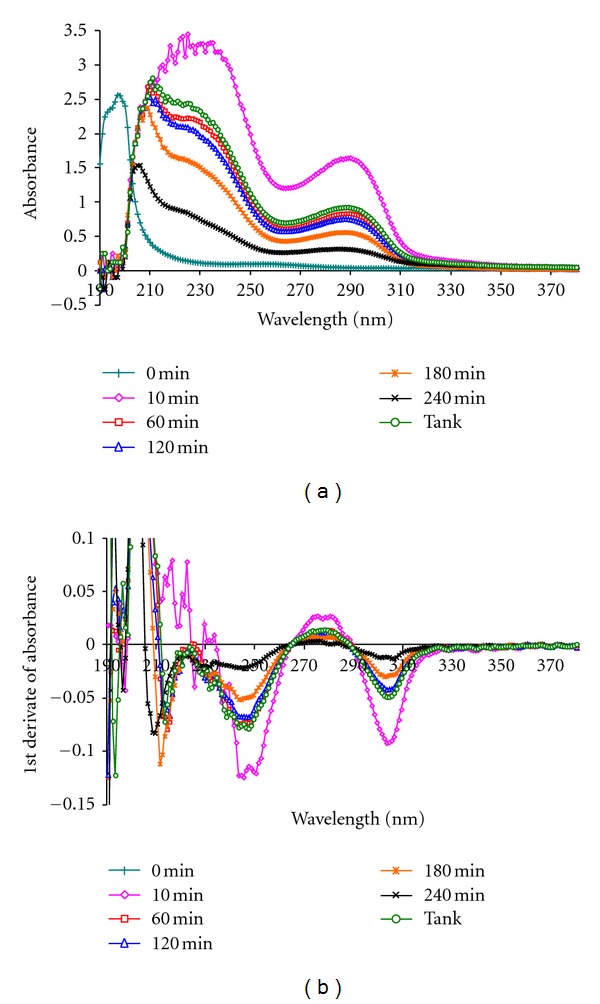
Example of absorbance spectrum (a) and first derivate of absorbance spectrum (b) obtained over wavelength range of 190–380 nm on spent dialysate samples at different times during dialysis session.

The obtained UV spectra were processed with a signal-processing tool using a Savitzky-Golay algorithm for smoothing, and the first derivative calculation wherein a smoothing window with nine points was used (Figure 2). Panorama Fluorescence 1.2 was used for signal processing, and multiple stepwise regression analysis was performed with Statistica 9.0. Final data processing was performed in EXCEL (Microsoft Office Excel 2007).

On the basis of the UA concentrations measured in the laboratory, measured UV absorbance spectra and processed UV absorbance spectra, multiple regression analysis was carried out on the calibration set of material (data from 75 randomly selected dialysis procedures). UA was set as a dependent variable, and UV absorbance values between 190–380 nm were set as independent variables. Multiple linear regression (MLR) analysis using the forward stepwise regression method was employed to determine the best wavelengths for the models [21–25]. Using the stepwise regression method helps us avoid mistakes in the models due to the possible collinearity of the independent variables [26]. In both UV absorbance (UVa) and the first derivate of UV absorbance (UVd), the number of steps was increased until no relevant improvements were achieved by means of model performance. At each step the model for estimation of UA was saved, resulting in different models for both UVa and UVd.

Models for the calculation of the concentration of UA (*Y*) are in the form

(1) $$Y=a+b_{1}*x_{1}+b_{2}*x_{2}+\cdots +b_{i}*x_{i},$$

where *a* is intercept, *b* is slope and *x* is an independent variable (the value of original or derivate UV absorbance at a certain wavelength).

The obtained models were used on the data from the remaining 113 dialysis procedures (validation set) to calculate the concentration of UA and compare these values with the laboratory results and validate different models.

Systematic error was calculated for the models as follows [26]:

(2) $$\text{BIAS}=\frac{\sum_{i=1}^{N}e_{i}}{N},$$

where *e*_*i*_ is the residual and *N* is the number of observations.

Standard error was calculated for the models as follows:

(3) $$\text{SE}=\sqrt{\frac{\sum_{i=1}^{N}{\left(e_{i}-\text{BIAS}\right)}^{2}}{N-1}}.$$

Root mean squared error was calculated for the models as follows:

(4) $$\text{RMSE}=\sqrt{\frac{\sum_{i=1}^{N}{\left(e_{i}\right)}^{2}}{N}}.$$

## 3. Results

**Figure 3:**
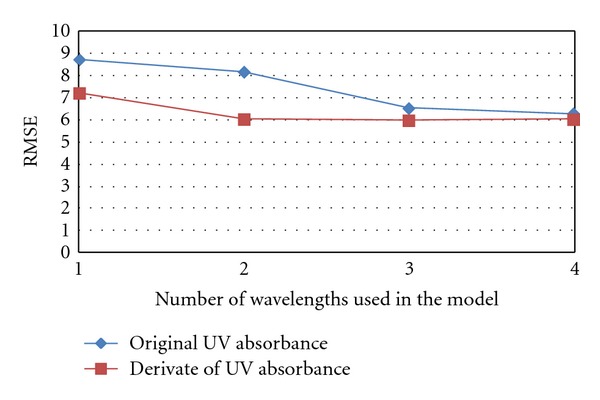
Behavior of RMSE with different models including 1–4 independent variables.

During regression analysis, three steps were considered sufficient after estimation of the behaviour of the root mean squared error (RMSE). From Figure 3 it was concluded that adding one additional wavelength to the models did not markedly improve the results in terms of RMSE. This was also confirmed by a *t*-test for residuals, which were significantly different (at *P* level 0.05) between models that used an absorbance or first derivate of absorbance value from one, two, or three wavelengths and which were not different in the case of models which used four wavelengths.

As a result of regression analysis, three models for UV absorbance and three models for derivate of UV absorbance were found wherein each used an absorption or derivate of absorption value from one, two, or three wavelengths, respectively (Table 4). The models were marked as UVa_1WL for the model which used a UV absorbance value from one wavelength, UVa_2WL for the same information from two wavelengths, and so on. UVd_1WL-UVd_3WL marks models which used a derivative value of UV absorbance from one, two, or three wavelengths.

**Figure 4:**
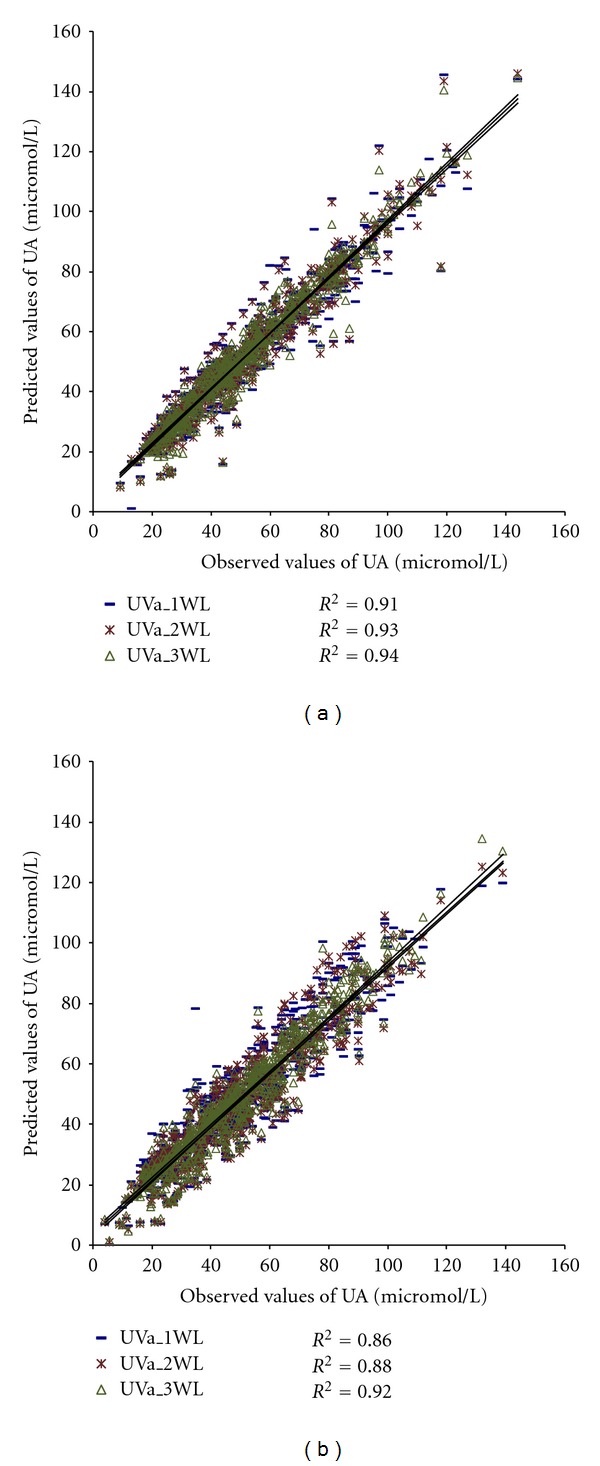
Models using UV absorbance values from one, two, or three wavelengths to estimate concentration of UA: (a) calibration group (*N* = 579) and (b) validation group (*N* = 639).

**Figure 5:**
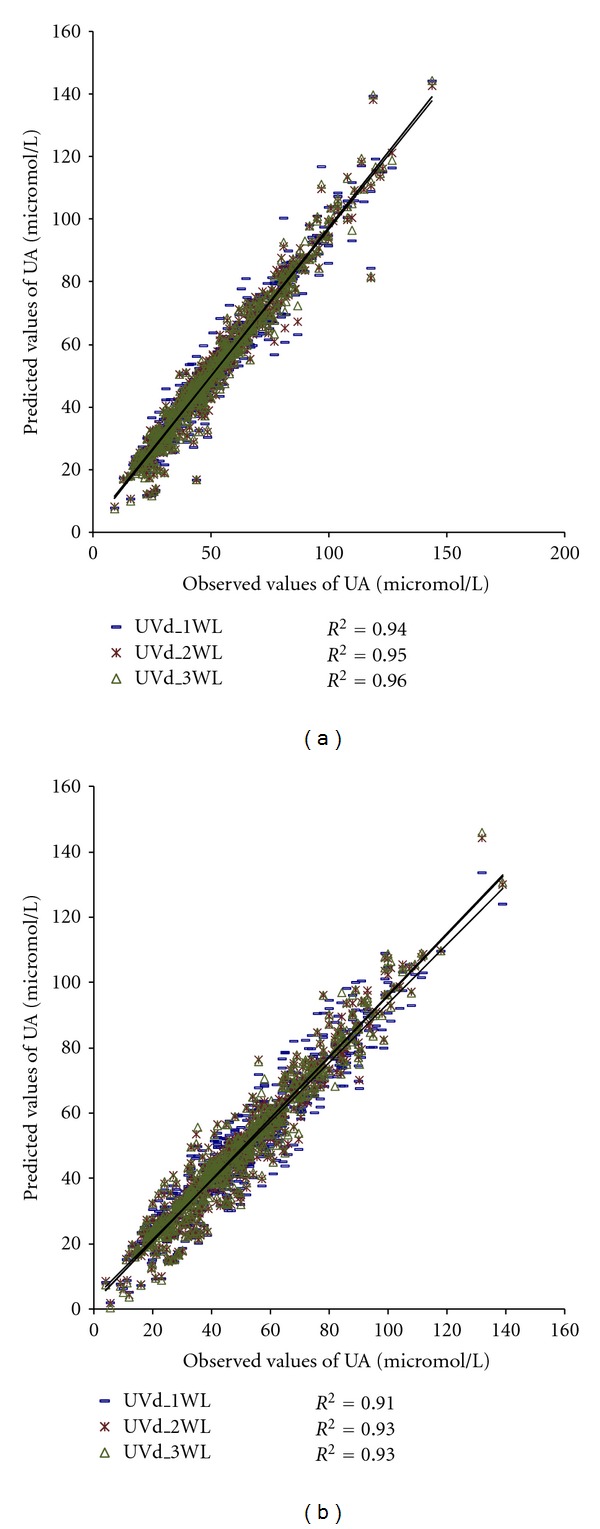
Models using values of first derivate of UV absorbance from one, two, or three wavelengths to estimate concentration of UA: (a) calibration group (*N* = 579) and (b) validation group (*N* = 639).

Figures 4 and 5 show the wavelengths of original UV absorbance and first derivate of UV absorbance included in the models for estimating UA concentration.

The models presented in Figures 4 and 5 were applied to the material to calculate UA concentrations, *R* ^2^, BIAS, SE, and RMSE. The results are presented in Table 5.

The concentrations achieved by the models were not significantly different (*P* = 0.17–0.48) from the observed concentrations in the laboratory for any model.

The systematic and root mean squared errors were significantly different (at *P* level 0.05) in the following cases (validation group):

- UVa_1WL versus UVd_1WL,
- UVa_1WL versus UVa_2WL,
- UVa_1WL versus UVa_3WL,
- UVd_1WL versus UVd_3WL,
- UVd_2WL versus UVd_3WL.

**Figure 6:**
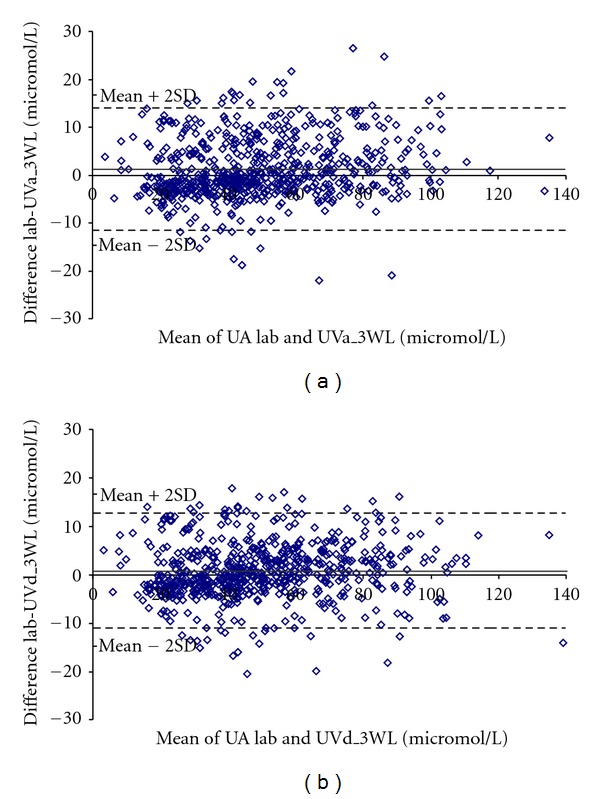
The Bland-Altman plots. (a) The difference between UA Lab and UVa_3WL is plotted against the mean value of UA Lab and UVa_3WL (*N* = 639). (b) The difference between UA Lab and UVd_3WL is plotted against the mean value of UA Lab and UVd_3WL (*N* = 639).

The differences between individual values of the UA concentration from the laboratory and UA values from two models (UVa_3WL and UVd_3WL) are presented in Figure 6.

The root mean squared error decreased as wavelengths were added to the models in the case of both the UVa and UVd models, and the decrease was slightly greater in the case of UVd models.

These results demonstrate that using UV absorbance from several wavelengths provides more accurate results in the estimation of the concentration of UA. Also, using information from the first derivate of spectra instead of original UV absorbance spectra produces a notable effect.

## 4. Discussion

The results in Table 5 show that it is possible to estimate UA concentration in spent dialysate using UV absorbance data. The presented models were built on the calibration set of material which contained absorbance values from Tallinn, Estonia, and Linköping, Sweden. The data included in the study were collected during seven studies from 1999 to 2009.

The coefficient of determination, *R* ^2^, between the laboratory and calculated values of UA are higher or equal in the case of the UVd (single/two/three) compared to the UVa (single/two/three) (0.86/0.88/0.92 versus 0.91/0.93/0.93) (Figures 4 and 5). Also, the systematic error and RMSE are lower if we use several wavelengths and/or derivate spectra (Table 5). This indicates that using several wavelengths instead of a single one produces a significant effect, which is larger when we use processed spectra instead of original absorbance spectra. However, it seems that adding a third wavelength to the UVd model does not improve results in terms of *R* ^2^, although the results of systematic error and RMSE improve. For describing the differences between individual values of the UA concentration from the laboratory and UA values from models, a Bland Altman plot for two models (UVa_3WL and UVd_3WL) was created (Figure 6); differences in UA values were somewhat smaller in the case of the model using derivate spectral values.

 Considering the improvement in the accuracy of the model, systematic error and RMSE, the signal processing and information from several wavelengths should be used in the future. In this study the best result was achieved with the model using derivate spectra values at three wavelengths.

It was found that haemodialysis adequacy can be quantified using UV absorbance of spent dialysate. By using this method, it is possible to reduce costs by reducing the number of blood samples and amount of laboratory analyses [27].

A good way of estimating UA concentrations using the UV technique has been shown in previous studies [13, 14, 16–20], but if we use signal processing tools and absorbance information from several wavelengths, we can essentially improve the accuracy and reliability of the results.

A previous study by our group [28] indicated that app. 90% of the cumulative and integrated UV absorbance measured by the optical dialysis adequacy sensor originates from the ten main peaks of a particular dialysis treatment, one of which is UA. Another study where HPLC analysis was used indicated that the main solute responsible for UV absorbance of around 280 nm is UA [29].

**Figure 7:**
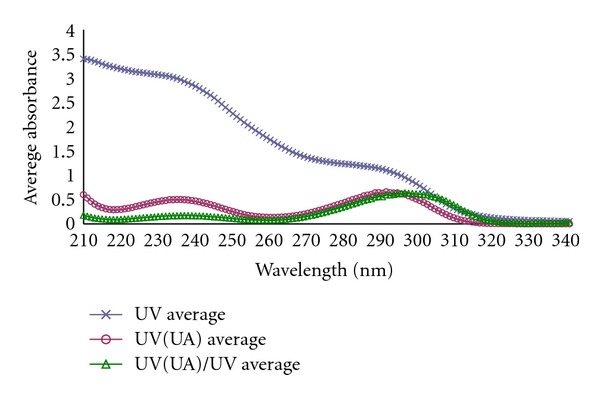
Average values of measured UV absorbance for total material and contribution of UA to UV absorbance.

As can be seen from Figure 7, the contribution of UA to total UV absorbance (UV (UA)/UV average presents an average absorbance sourced from UA in the dialysate divided by average UV absorbance of the whole dialysate) is relatively large in the wavelength region of 280–310 nm. This explains the wavelengths appearing in the models. UA absorbance spectra have one minimum around 265, and this explains why the wavelength is also included in the models.

The high correlation between UV absorbance and UA could be explained by the characteristic absorbance around 294 nm for UA in combination with the relatively high molar extinction coefficients of UA in this wavelength region compared to other chromophores among uremic retention solutes eliminated from blood into spent dialysate during dialysis [30]. This makes it possible to determine UA concentration even when the technique does not solely measure UA.

The use of a Savitzky-Golay algorithm for smoothing and first derivate calculation is an effective method of correcting baseline effects in spectra, which could explain the improvement in accuracy. Using UV absorbance and processed UV absorbance information from several wavelengths reduces randomness and is probably the reason why better results have been achieved.

In this study, multiple linear regression (MLR) analysis using the forward stepwise regression method was used to determine the best wavelengths for models. Using the stepwise regression method helps us to avoid mistakes in the models due to the possible collinearity of independent variables. It seems that models developed with MLR are relevant and work well in a validation set of material, although using other approaches like partial least squares regression (PLS-R) or principal component regression (PCR) to create models should be considered in the future [26].

The clinical aim in the future is to develop an online monitoring system that offers an estimation of the removal of clinically important solute and marker UA during haemodialysis.

Also, regarding the optical properties of UA, it is possible to develop an optical system to measure the UA concentration in blood and/or urine. This makes it possible to rapidly detect hyperuricemia widely and at an early stage. This is very important in preventing serious clinical issues caused by hyperuricemia [2–6, 8, 11, 12, 31].

An accurate optical method makes it possible to measure UA rapidly online without the need for blood samples and disposables or chemicals. Using a simple signal-processing tool and UV absorbance values from several wavelengths could be very helpful in achieving more accurate and reliable results.

## 5. Conclusion

This study investigated the effect of using several wavelengths and a simple signal processing to estimate the concentration of UA in dialysate using an optical method. The data analysed were collected over 10 years: 60 patients participated and 188 dialysis sessions were monitored in various centres in different countries. It was found that using a multi-wavelength and processed signal approach leads to more accurate results. This approach enables us to develop an advantageous, reliable, and cost-effective method of measuring the concentration of UA, an independent risk marker of cardiovascular and renal diseases and also a novel risk factor for type 2 diabetes mellitus. Developed algorithms could be used in optical dialysis quality monitors; these monitors should be integrated to dialysis machines and with these several parameters; UA among them is possible to monitor during the dialysis. No blood will be monitored; removal on substances is possible to estimate only by monitoring the spent dialysate. A future method evaluates the treatment dose and makes it possible to control treatments against set target values.

## Figures and Tables

**Table 1:** Summary of the of the studies and patients participated.

Study	No. of sessions	No. of patients (male/female)	Mean age
1	40	10 (6/4)	63 ± 21
2	19	7 (4/3)	57 ± 23
3	40	10 (6/4)	60 ± 19
4	30	10 (7/3)	63 ± 19
5	11	7 (4/3)	56 ± 13
6	24	8 (7/1)	77 ± 7
7	24	8 (7/1)	77 ± 7

**Table 2:** Summary of the conditions of the studies.

Study	Dialyser	Area, m^2^	*N*	Dialysis machine	Blood flow, mL/min
1	AF180	1.8	40	AK200 Fresenius 4008 H	250–300
2	AF180	1.8	7	AK200	300–350
	Polyflux17S	1.7	12	Fresenius 4008 H	
3	Polyflux17L	1.7	18	AK200	200–350
	TCA150G	1.5	3	Fresenius 4008 H	
	Nephral300	1.3	9		
4	F8	1.8	14	Fresenius 4008 H	245–350
	F10	2.2	3		
	FX80	1.8	13		
5	FX80	1.8	11	Fresenius 4008 H	245–350
6	FX80	1.8	24	Fresenius 5008	280–350
7	FX800	1.8	24	Fresenius 5008	280–350

**Table 3:** Summary of the samples taken during the studies.

Study	Sampling time, min.
1	5, 15, 30, 60, 90, 120, 180, 240, 270, 300, tank
2	5, 15, 30, 60, 90, 120, 180, 240, 255, 270, 300, tank
3	5, 60, 120, 180, 240, tank
4	10, 60, 120, 180, 240, tank
5	10, 60, 120, 180, 240
6	10, 240, tank
7	10, 30, 60, 120, 180, 240, 270, tank

**Table 4:** Summary of achieved models.

Model for	*a*	*b*_1_∗*x*_1_	*b*_2_∗*x*_2_	*b*_3_∗*x*_3_
original UV absorbance spectra at 294 nm (UVa_1WL)	−2.28	51.69∗A_294_		
original UV absorbance spectra at 294 and 312 nm (UVa_2WL)	−1.67	60.56∗A_294_	−60.75∗A_312_	
original UV absorbance spectra at 294, 312 and 266 nm (UVa_3WL)	−1.55	75.38∗A_294_	−62.27∗A_312_	–7.36∗A_266_
derivative spectra at 300 nm (UVd_1WL)	−1.44	−1038.84∗D_300_		
derivative spectra at 300 and 270 nm (UVd_2WL)	−2.12	−1111.09∗D_300_	128.67∗D_270_	
derivative spectra at 300, 270 and 222 nm (UVd_3WL)	−3.56	−1128.73∗D_300_	120.74∗D_270_	−32.54∗D_222_

**Table 5:** Summary of results of different methods of measuring concentration of uric acid.

Method	Set	*N*	Concentration of UA ± SD (micromol/L)	*R* ^2^	BIAS	SE	RMSE
Lab	Cal.	579	52.1 ± 23.3	—	—	—	—
	Val.	639	49.7 ± 23.0	—	—	—	—

UVa_1WL	Cal.	579	52.1 ± 22.3	0.91	0.00	6.83	6.83
	Val.	639	48.9 ± 21.8	0.86	−0.88	8.70^a,b,c^	8.74^a,b,c^

UVa_2WL	Cal.	579	52.1 ± 22.5	0.93	0.00	6.19	6.19
	Val.	639	48.1 ± 21.6	0.88	−1.70	8.00	8.18

UVa_3WL	Cal.	579	52.1 ± 22.6	0.94	0.00	5.52	5.52
	Val.	639	48.4 ± 21.8	0.92	−1.39	6.39	6.54

UVd_1WL	Cal.	579	52.1 ± 22.6	0.94	0.00	5.64	5.64
	Val.	639	48.2 ± 21.8	0.91	−1.57	7.05^d^	7.22^d^

UVd_2WL	Cal.	579	52.1 ± 22.8	0.95	0.00	4.95	4.95
	Val.	639	48.7 ± 22.3	0.93	−1.07	5.94^e^	6.04^e^

UVd_3WL	Cal.	579	52.1 ± 22.8	0.96	0.00	4.83	4.83
	Val.	639	48.9 ± 22.4	0.93	−0.89	5.92	5.99
